# Evolutionary Convergence of Nutritional Symbionts in Ticks

**DOI:** 10.1111/1758-2229.70120

**Published:** 2025-06-10

**Authors:** Noor Fattar, Meriem Louni, Marie Buysse, Anna Maria Floriano, Joanne Bertaux, Anne Cantereau, Ana Rivero, Marjorie Bruley, Karen D. McCoy, Vincent Delafont, Nathalie Boulanger, Fabrice Vavre, Didier Bouchon, Olivier Duron

**Affiliations:** ^1^ MIVEGEC University of Montpellier Centre National de la Recherche Scientifique (CNRS), Institut de Recherche Pour le développement (IRD) Montpellier France; ^2^ Centre National de la Recherche Scientifique (CNRS) UMR 7267, EBI, University of Poitiers Poitiers France; ^3^ Université Claude Bernard Lyon 1 UMR 5558, LBBE, CNRS, VetAgroSup Villeurbanne France; ^4^ UAR CNRS 2038 BioSanté, ImageUP Université de Poitiers Poitiers France; ^5^ UR3073: PHAVI: Pathogen‐Host‐Arthropod Vector Interactions, Groupe Borréliose de Lyme Université de Strasbourg Strasbourg France; ^6^ Centre National de Référence Borrelia – Hôpitaux Universitaires de Strasbourg Strasbourg France

## Abstract

Symbiosis with bacteria is essential for the survival of animals with an obligate blood‐feeding lifestyle. In ticks, two distinct bacterial lineages, *Coxiella*‐like and *Francisella*‐like endosymbionts, have independently evolved into nutritional symbionts, converging on a key biochemical function for the tick's survival and growth: the production of three B vitamins. In this study, we carried out comparative analyses across multiple tick species and characterised remarkable similarities in their tissue localisation, particularly in organs important for nutrient metabolism and maternal transmission to progeny. In these organs, both symbionts colonise similar intracellular niches, residing within membrane‐bound, replicative vacuoles that occupy a substantial part of the cytoplasm of tick cells. Despite extensive genomic reduction, both symbionts have retained pathways for the biosynthesis of B vitamins and, in some cases, chorismate, a precursor used for the production of serotonin by ticks. However, differences exist: while *Coxiella*‐like endosymbionts lack the ability to synthesise heme, *Francisella*‐like endosymbionts possess a complete heme biosynthesis pathway and may potentially provide ticks with this essential cofactor. Overall, these phenotypic and genomic characteristics reveal a broad convergence among symbiotic interactions across major tick families, highlighting the essential role of symbiosis in tick nutrition, feeding behaviour, blood intake and subsequently in pathogen transmission.

## Graphical abstract

Confocal imaging of nutritional symbionts in the ovaries of ticks using fluorescence in situ hybridisation.

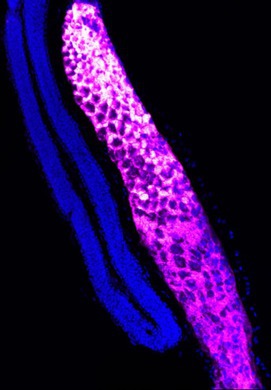

## 1 Introduction

Blood feeding is one of the most specialised and metabolically challenging diets observed in the animal kingdom (Duron and Gottlieb 2020; Manzano‐Marín et al. 2015; Nikoh et al. 2014). Blood, although rich in proteins, iron, and salts, is critically deficient in carbohydrates, lipids, and essential vitamins (Sterkel et al. 2017). Obligate blood feeders, such as ticks, leeches, and bed bugs, rely exclusively on blood throughout their entire life cycle and have evolved key adaptations for haemoglobin digestion, iron homeostasis, and compensating lipid and vitamin deficiencies (Babenko et al. 2020; Benoit et al. 2016; Jia et al. 2020; Gulia‐Nuss et al. 2016). Obligate blood feeders have also converged on analogous functional microbiomes, dominated by bacterial symbionts that can synthesise the B vitamins necessary for their survival, growth, and reproduction (Duron and Gottlieb 2020; Manzano‐Marín et al. 2015; Nikoh et al. 2014; Cornwallis et al. 2023). Indeed, bacterial symbionts are metabolically more diverse than animals and can produce essential amino acids and vitamins that their hosts cannot (Duron and Gottlieb 2020; Manzano‐Marín et al. 2015; Nikoh et al. 2014; Cornwallis et al. 2023). Through this synergistic interplay of genomic adaptations and microbiome innovations, distinct animal lineages have successfully exploited blood as an exclusive nutritional resource, driving their ecological expansion and enhancing their evolutionary diversification (Duron and Gottlieb 2020; Cornwallis et al. 2023).

Ticks are obligate hematophagous arachnids, comprising over 900 species that rely exclusively on blood as their sole source of nutrition throughout all stages of their life cycle (Sonenshine and Roe 2014). They harbour complex external (cuticular) microbiota, including diverse bacteria and fungi, whose communities are influenced by factors such as geographic origin, natural habitat, and host associations (Binetruy et al. 2019; Pollet et al. 2020; Bruley et al. 2025). In contrast, ticks typically harbour low‐complexity internal microbiomes, largely dominated by B vitamin–provisioning symbionts, which coexist with a few other bacterial taxa and, when present, pathogens, with composition varying by tick species (Duron and Gottlieb 2020; Bonnet and Pollet 2021). Most tick species harbour B vitamin–provisioning Legionellales symbionts, commonly referred to as *Coxiella*‐like endosymbionts (CLE). In contrast, species that lack CLE typically host alternative B vitamin–provisioning symbionts belonging to distantly related bacterial orders, such as Thiotrichales symbionts known as *Francisella*‐like endosymbionts (FLE), or other unrelated symbiotic bacteria (Duron and Gottlieb 2020; Amoros et al. 2024). These intracellular symbionts have been identified across various genera of both soft ticks (Argasidae) and hard ticks (Ixodidae) (Duron et al. 2017; Azagi et al. 2017; Binetruy et al. 2020; Buysse et al. 2021; Lalzar et al. 2012; Clay et al. 2008; Machado‐Ferreira et al. 2011; Duron, Noël, et al. 2015), despite earlier misidentifications as tick‐borne pathogens (Duron 2015; Buysse and Duron 2021; Duron, Sidi‐Boumedine, et al. 2015). Surveys of tick microbiota have consistently reported an exclusion pattern between CLE and FLE across tick species hosting these symbionts: their distribution is non‐random, with most tick species harbouring either CLE or FLE, and their co‐presence in the same species being rare (significantly less frequent than would be expected by chance), thus underscoring a clear exclusion pattern (Duron et al. 2017; Binetruy et al. 2020). Phylogenetic evidence suggests that CLE‐FLE symbiotic interactions in ticks are dynamic, involving multiple events of losses and replacements (Duron et al. 2017; Binetruy et al. 2020; Gerhart et al. 2018; Buysse et al. 2022), and possibly several independent transitions to mutualism for CLEs (Santos‐Garcia et al. 2023; Brenner et al. 2021).

Although CLE and FLE are phylogenetically distant bacteria, they have independently converged to a similar B‐vitamin‐based nutritional mutualism (Duron and Gottlieb 2020). When deprived of their CLE or FLE symbionts, tick nymphs exhibit severe developmental issues, including physical abnormalities, cessation of feeding, and failure to moult (Ben‐Yosef et al. 2020; Li et al. 2018; Duron et al. 2018; Zhong et al. 2007; Guizzo et al. 2017), that can be restored by artificial supplementation of B vitamins (Duron et al. 2018). Genome sequencing of CLE and FLE has revealed complete biosynthetic pathways for three essential B vitamins: biotin (B_7_), riboflavin (B_2_), and folate (B_9_). In contrast, other B vitamin synthesis pathways are partially degraded or absent in most genomes (Gerhart et al. 2018, 2016; Santos‐Garcia et al. 2023; Brenner et al. 2021; Duron et al. 2018; Guizzo et al. 2017; Gottlieb et al. 2015; Nardi et al. 2021; Smith et al. 2015; Tsementzi et al. 2018) with only a few exceptions (Buysse et al. 2021). Reflecting their reliance on nutritional symbionts, ticks have evolved specific traits that stabilise these interactions. For example, despite their distinct phylogenetic origins, both CLE and FLE rely on maternal (transovarial) transmission for their persistence within tick populations (Machado‐Ferreira et al. 2011; Duron, Noël, et al. 2015; Duron et al. 2018; Lalzar et al. 2014).

The extent to which similarities among symbiotic interactions with CLE and FLE extend beyond their B‐vitamin‐based nutritional symbiosis remains largely unexplored. Interestingly, CLE and FLE are closely related to virulent intracellular pathogens of humans and other mammals: CLE with *Coxiella burnetii* , the causative agent of Q fever, and FLE with *Francisella tularensis* , responsible for tularemia (Duron, Noël, et al. 2015; Duron et al. 2018). Both pathogens primarily infect mammalian macrophages, where they replicate within specialised cytoplasmic compartments that they manipulate to create a replication niche (van Schaik et al. 2013; Petit and Lebreton 2022; Degabriel et al. 2023). In the tick *Rhipicephalus turanicus* , CLE are predominantly packed in small intracellular vesicles visually similar to the *C. burnetii* ‐containing vacuole observed in mammals (Lalzar et al. 2014). However, similar investigations of CLE and FLE in other tick species have not been conducted, resulting in significant gaps in our knowledge regarding these symbiotic interactions. The same holds true for additional key traits of the symbiosis, which have been observed only once or rarely for CLE, and never for FLE. For instance, in the Asian long‐horned tick *Haemaphysalis longicornis* , CLE was found to possess a complete shikimate pathway for the biosynthesis of chorismate, a precursor used by ticks to produce tryptophan and serotonin, which in turn influences tick feeding behaviour and blood intake (Zhong et al. 2021). In several tick species, CLE was found to be abundant in the Malpighian tubules (Duron et al. 2018; Guizzo et al. 2017; Lalzar et al. 2014; Wang et al. 2018), where it may utilise nitrogenous wastes to synthesise B vitamins (Duron and Gottlieb 2020). In contrast, the presence of FLE in the Malpighian tubules has been documented in only one study in the soft tick *Ornithodoros moubata* (Duron et al. 2018), and the generality of FLE presence in this organ remains unknown.

In this study, we explore the hypothesis of broader evolutionary convergences in nutritional symbionts in ticks, encompassing not only B‐vitamin synthesis but also broader symbiotic mechanisms found across different tick species. To this aim, we examined the tissue distribution and cellular phenotypes of CLE and FLE across a range of soft and hard tick species. We also compared the genomic content of CLE and FLE in these tick species and looked for key biosynthetic pathways involved in symbiotic interactions. Our results confirm that the evolutionary convergence between CLE and FLE symbionts extends beyond their apparent B‐vitamin‐based nutritional symbioses.

## 2 Material and Methods

### 2.1 Tick Specimens and Organ Collection

We examined adult males and females belonging to two related species of soft ticks, *Ornithodoros maritimus* and *Ornithodoros moubata* , and two closely related species of hard ticks, *Dermacentor marginatus* and *Dermacentor reticulatus* . These species naturally harbour either CLE ( *O. maritimus* , *D. marginatus* ) or FLE ( *O. moubata* , *D. reticulatus* ) as nutritional symbionts (Duron et al. 2017, 2018, 2014; Duron, Noël, et al. 2015; Buysse et al. 2022; Gomard et al. 2021). Ticks were either collected from natural populations or from laboratory colonies (Table S1).

Each tick specimen was thoroughly washed in three consecutive baths of sterile water to remove any external environmental contamination. Using sterile blades and forceps, we dissected the tick organs (reproductive organs, Malpighian tubules, midgut, and salivary glands) under a stereomicroscope. Prior to further processing, all dissected organs and tick carcasses were rinsed with sterile saline solution, following the specific protocols detailed in subsequent sections.

### 2.2 Molecular Quantification of Tick Symbionts

CLE and FLE densities were assessed at the organ and carcass level from unfed females (F) and males (M) of the four tick species: *O. maritimus* (n_F_ = 6, n_M_ = 4), *O. moubata* (n_F_ = 5, n_M_ = 3), *D. marginatus* (n_F_ = 8, n_M_ = 4), and *D. reticulatus* (n_F_ = 6, n_M_ = 5). DNA was extracted from organs and carcasses using the DNeasy Blood and Tissue Kit (QIAGEN, Hilden, Germany), following the manufacturer's instructions. Each DNA extract was then tested by a quantitative polymerase chain reaction (qPCR) assay using the SYBR Green Master Mix (Applied Biosystems, Waltham, Massachusetts, USA). Two PCRs were performed for each organ and carcass: one specific for the CLE or FLE *rpoB* gene, and the other specific for the tick *Act2* gene (Table S2). Assuming that both genes are present in a single copy per haploid genome of the tick and the symbiont, the ratio between *rpoB* and *Act2* concentrations estimates the number of symbiont genomes relative to the number of tick genomes, thus correcting for tick organ or carcass size. Each DNA template was analysed in triplicate for *rpoB* and *Act2* quantification. Standard curves were plotted using dilutions of a pEX‐A2 vector (Eurofins, Ebersberg, Germany) containing one copy of each of the *rpoB* and *Act2* gene fragments. CLE densities of *O. maritimus* and *D. marginatus* , and FLE densities in *D. reticulatus* were acquired with a LightCycler96 (Roche, Basel, Switzerland). FLE densities of *O. moubata* ticks were previously acquired with a LightCycler480 (96) (Roche) (Duron et al. 2018).

### 2.3 Statistical Analyses

Differences in symbiont densities were analysed using R (v4.2.2), implemented in RStudio (v2023.06.1) (https://www.r‐project.org). Data normality was assessed by the Shapiro–Wilk test, and goodness‐of‐fit was evaluated using the *fitdistrplus* package (Delignette‐Muller and Dutang 2015). Based on these results, log‐transformed symbiont densities were analysed using generalised linear mixed models (GLMM) with a Gaussian error distribution (*glmer* function, *lme4* package). Tick organ, sex, and symbiont type were included as fixed effects, while individual tick, qPCR plate, and operator were included as random effects to account for data heterogeneity and repeated measures. Model simplification was performed by sequentially removing explanatory variables and their interactions to identify the minimal adequate model, using ANOVA for model comparison (*anova* function, *car* package (Fox and Weisberg 2019)). Model diagnostics, including residuals and fit quality, were assessed using the DHARMa package (Hartig 2022). *Post hoc* pairwise comparisons were conducted, and least square means were calculated using the *emmeans* package (*emmeans* function (Lenth 2022)), with Tukey's correction for multiple testing. Two types of analyses were carried out: (i) an analysis that included all reproductive organs except ovaries and testes (o avoid potential bias from sex‐specific effect) and (ii) analyses separately for each organ (datasets stratified by organ to account for organ‐specific effects on symbiont distributions).

### 2.4 Fluorescence In Situ Hybridization

The localization of CLE and FLE within tick ovaries was visualised using fluorescence in situ hybridization (FISH). Dissected ovaries from each tick species were fixed immediately and processed according to the protocol described by Amoros and Fattar et al. (Amoros et al. 2024), with adjustments applied to paraformaldehyde (PFA) fixation and washing times tailored to each tick species. For *O. moubata* , ovaries were initially fixed for 1 h, washed for 15 min, fixed a second time for 30 min and then rinsed twice for 15 min each. For *O. maritimus* , ovaries were fixed for 1 h and rinsed twice for 15 min each. For *D. marginatus* and *D. reticulatus* , the fixation time of ovaries was adjusted to 30 min and washing was performed twice for 15 min each. Specific 16S rRNA‐specific probes were designed for each tick species and symbiont (Table S3). Hybridization with the FLE‐specific probe (Frla_F2) included two probe helpers (Table S3). Negative controls were obtained by incubating additional ovaries without CLE and FLE‐specific probes, followed by assessment of tissue autofluorescence. FISH images were then acquired using an Olympus FV3000 confocal microscope at the ImageUP facility at the University of Poitiers and analysed following Amoros and Fattar et al. (Amoros et al. 2024).

### 2.5 Transmission Electron Microscopy

Detailed images of CLE and FLE within tick ovary cells were obtained using Transmission Electron Microscopy (TEM). After dissection, ovaries were incubated in fixation buffer (glutaraldehyde 9%, sodium cacodylate 0.3 M, NaCl 3%, v/v/v, pH 7.4) for 2 h. Following fixation, four washes of 10 min each were performed with washing buffer (sucrose 0.6 M, sodium cacodylate 0.1 M, v/v) to remove the fixative solution. Subsequently, the samples were incubated in a novel buffer (osmium tetroxide 3.9%, sodium cacodylate 0.3 M, sucrose 0.75 M, v/v/v) for 1 h in the dark at room temperature. Dehydration was carried out through a graded series of acetone washes (50% [2 × 1 min], 70% [2 × 5 min], 90% [2 × 15 min], 100% [4 × 15 min], with each step lasting 15 min). The samples were then incubated in a 1:1 mixture of acetone and resin (Polysciences Inc., USA) for 12 h. They were then embedded in pure resin and cured in an oven at 70°C for 24 h. After polymerisation, the blocks were cut into ultrathin sections (70 nm) with an EMUC6 Leica ultramicrotome. The sections were contrasted with 2% uranyl acetate and lead citrate. Specimens were viewed using a Jeol JEM 1010 electron microscope with a Quemesa Olympus digital camera at the ImageUP facility at the University of Poitiers. A TEM visualisation of symbionts within tick cells was then successfully obtained for *O. maritimus* , *D. marginatus* , and *O. moubata* . Unfortunately, due to a fixation issue, high‐quality tissue samples could not be obtained for *D. reticulatus* , preventing FLE visualisation for this species.

### 2.6 Symbiont Genomes

The metagenome‐assembled genome (MAG) of *D. reticulatus* was obtained from a DNA extraction enriched for FLE DNA following Duron et al. (Duron et al. 2018). Malpighian tubules and ovaries were dissected from three adult *D. reticulatus* ticks, pooled, and homogenised in 100 μL sterile double‐distilled water. The resulting homogenate was diluted in 10 mL sterile double‐distilled water and incubated for 1 h at 20°C. To remove tick nuclei and other cellular debris, the homogenate was filtered using a 5 μm Minisart filter (Sartorius, Göttingen, Germany). The remaining cells in the homogenate were pelleted by centrifugation at 20,000 × g for 15 min at 4°C. FLE‐enriched DNA was extracted from the pellet using the NucleoSpin Soil kit (Macherey‐Nagel, Düren, Germany). FLE‐enriched DNA was further quantified using a Qubit fluorometer with the dsDNA High Sensitivity Kit (Invitrogen, Carlsbad, California, USA). The library was prepared using the Nextera DNA Flex kit (Illumina, San Diego, California, USA) and subsequently sequenced on the Illumina NovaSeq 600 platform at the MGX DNA Services Facility in Montpellier, France. A total of 23,783,157 paired‐end reads were generated, which were then trimmed using Trimmomatic (v0.40) (Bolger et al. 2014) and assembled with SPAdes (v3.10.0) (Bankevich et al. 2012), following a modified Blobology pipeline (Kumar et al. 2013) as detailed on GitHub (https://github.com/annamariafloriano/EvoNutrSymb_Fattar_Louni). Contaminating sequences were discarded, and the assemblies were refined through manual analysis of the assembly graphs using Bandage (v0.8.1) (Wick et al. 2015). This process allowed for the separation of bacterial reads from tick reads, facilitating the assembly of the FLE genome, hereafter referred to as FLEDreti100‐P. The quality assessment and completeness of the assembly were evaluated using miComplete (v1.1.1) using Bact105 weights (Hugoson et al. 2020). Using this method, the FLEDreti100‐P genome was recovered as 36 contigs, resulting in an estimated size of 1,627,125 bp and completeness over 97% (Table S4).

The CLE genomes of *O. maritimus* (CLEOmar strain; GenBank: GCA_907164965.1) and *D. marginatus* (CLEDm strain; GenBank: GCA_907164955.1), as well as the FLE genome of *O. moubata* (F‐Om strain; GenBank: GCA_003069505.1), had been previously obtained following a similar protocol (Santos‐Garcia et al. 2023; Duron et al. 2018) using tick specimens collected from the same localities and laboratory colonies as the current study (Table S4).

### 2.7 Comparative Genomics

The two CLE genomes (CLEOmar and CLEDm) and the two FLE genomes (F‐Om and FLEDreti100‐P) were further compared using the genomes of *Coxiella burnetii* (RSA 493 strain; GenBank: GCA_000007765.2) and *Francisella tularensis* (SCHU‐S4 strain; GenBank: AJ749949). The CLE and FLE whole‐genome alignments were obtained separately using Mauve (Darling et al. 2004). All genomes were re‐annotated using Prokka (v1.14.6) with the compliant option (Seemann 2014). For each genome, intact genes and pseudogenes were distinguished using Pseudofinder (v1.1.0) (Syberg‐Olsen et al. 2022), followed by an analysis with BlastKoala (v3.0) (Kanehisa et al. 2016) to identify and compare metabolic pathways.

## 3 Results

### 3.1 Common Tissue Tropism of CLE and FLE Across Tick Species

**FIGURE 1:**
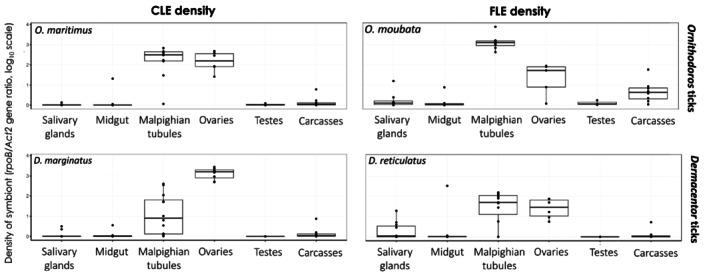
Boxplot of CLE and FLE densities according to tick species, sex, and organ. Symbiont densities (log_10_ scale) were quantified by calculating the ratio of CLE or FLE *rpoB* gene copies per tick *Act2* gene copy.

Real‐time qPCR revealed a distinct tissue tropism of symbionts towards the Malpighian tubules and ovaries, regardless of the symbiont type or tick species (Figure 1). These two organs exhibited significantly higher CLE and FLE densities compared to other tick organs and carcasses. The symbiont density in female ovaries was 70x higher than in male testes (mean ± s.e: 2.11 ± 0.18 and 0.03 ± 0.02, respectively; Model #1, *χ* ^2^_5_ = 115.38, *p <* 0.0001; Table S5), and 10‐14x higher than in any other organs (salivary glands: 0.15 ± 0.05; midgut: 0.15 ± 0.08) and the carcasses (0.22 ± 0.06) (Model #2, *χ* ^2^_5_ = 198.85, all *p* < 0.0001) except the Malpighian tubules (1.85 ± 0.17) (Model #2, *χ* ^2^_5_ = 1.536, *p* = 0.6413; Table S5). Despite converging distribution patterns in CLE and FLE densities, the GLMMs also revealed significant differences (Figure 1). In the ovaries, CLE had significantly higher densities than FLE (CLE: 2.70 ± 0.16, FLE: 1.36 ± 0.18; Model #3, *χ* ^2^_5_ = 39.627, *p* < 0.0001), whereas in the Malpighian tubules, the reverse occurred (CLE: 1.60 ± 0.13; FLE: 2.15 ± 0.14; Model #3, *p* = 0.0038; Table S5). A limitation of this measurement is that, while using a nuclear tick gene for qPCR assumes diploid tissues, Malpighian tubules and salivary glands harbour polytene chromosomes in various arthropods (Strashnyuk et al. 2023; Buntrock et al. 2012) although they have not yet been documented in ticks. This could theoretically lead to a single copy nuclear gene being amplified into multiple copies per arthropod cell, potentially affecting inter‐tissue comparisons. However, even if such a bias exists in ticks, it is unlikely to influence between‐species comparisons involving the same tissue type. Moreover, the strong symbiont signal observed in Malpighian tubules suggests that any potential bias due to polytene chromosomes would most likely lead to an underestimation, rather than an overestimation, of symbiont abundance.

### 3.2 Similarities of CLE and FLE Within Tick Tissues and Cells

**FIGURE 2:**
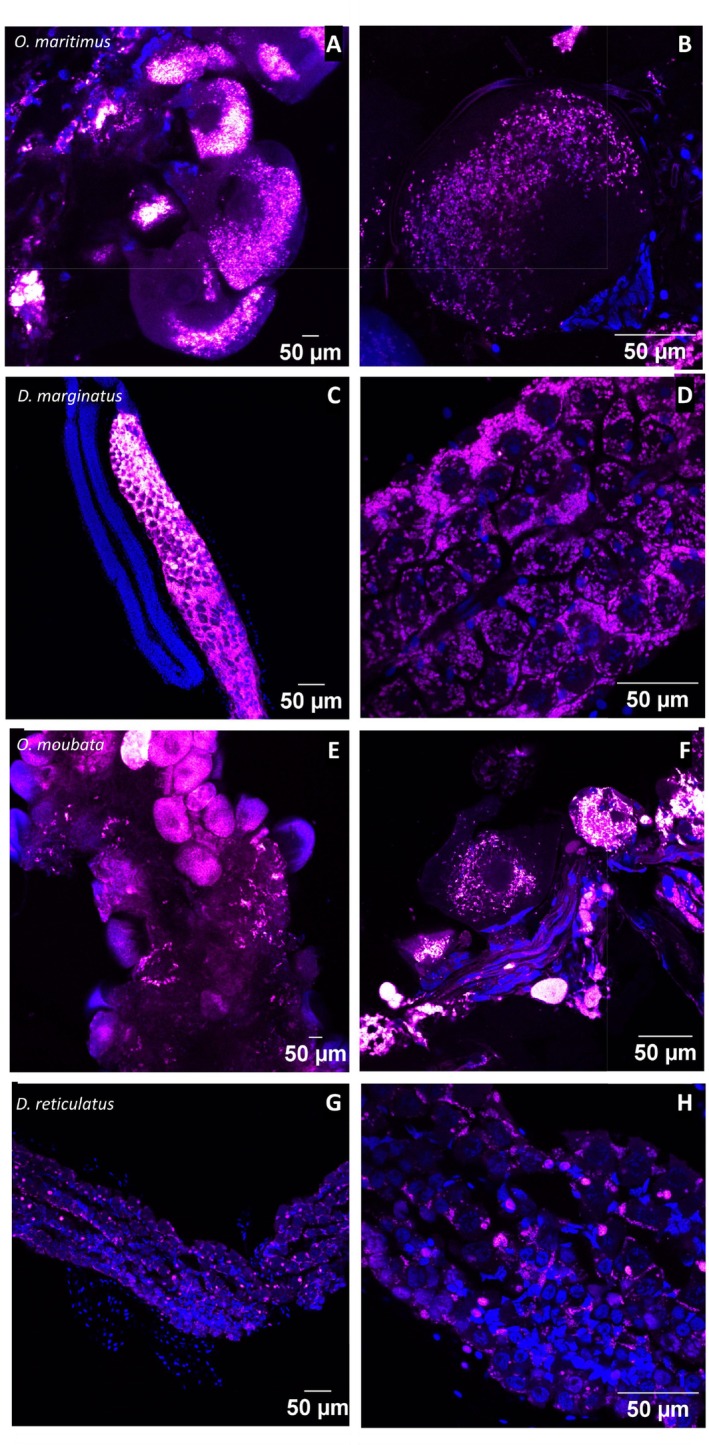
Confocal imaging of CLE and FLE in the ovaries of ticks using FISH. DNA was directly stained using DAPI (blue), and symbionts with specific probes (magenta). (A, B) *O. maritimus* and CLE; (C, D) *D. marginatus* and CLE; (E, F) *O. moubata* and FLE; (G, H) *D. reticulatus* and FLE.

**FIGURE 3:**
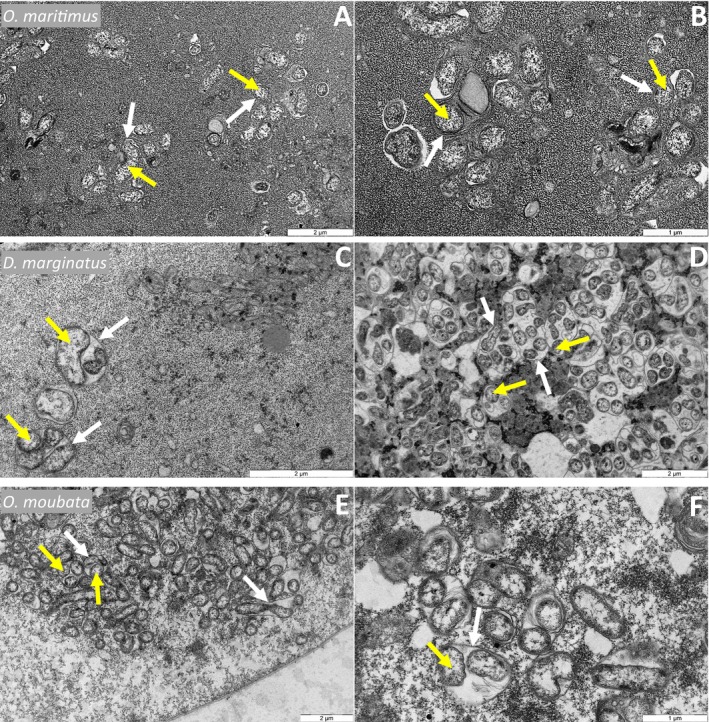
TEM micrographs of CLE and FLE in the ovaries of ticks. Transverse sections reveal double membraned vacuoles (white arrows) highly populated by symbionts (yellow arrows). (A, B) Stage III oocyte of *O. maritimus* and CLE; (C, D) Stage I oocyte of *D. marginatus* and CLE; (E, F) Stage III oocyte of *O. moubata* and FLE.

In accordance with the qPCR results, FISH and TEM analyses support the important presence of CLE and FLE in the ovaries of their respective tick species (Figures 2 and 3). In the ovaries, the symbionts filled a large portion of the volume in most developing oocytes across the four tick species. Higher magnification of the oocyte cells showed that CLE and FLE were enclosed in very similar small vacuoles, which collectively gave the cytoplasmic content a characteristic cottony appearance (Figure 2). A three‐dimensional FISH reconstruction of the symbionts' cytoplasmic distribution further revealed that these vacuoles were spread throughout the depth of the entire tick cell (Video S1). TEM analyses revealed cells densely populated with symbionts, neatly packed in small, double‐membraned vacuoles, each containing more than one bacterium (Figure 3). These vacuoles consistently exhibited a similar structure and size (1–2 μm), regardless of the symbiont type or tick species. Inside these vacuoles, dividing bacteria were often observed, suggesting ongoing CLE and FLE proliferation (Figure S1).

### 3.3 (c) Biosynthetic Pathway Overlaps in CLE and FLE Genomes

**FIGURE 4:**
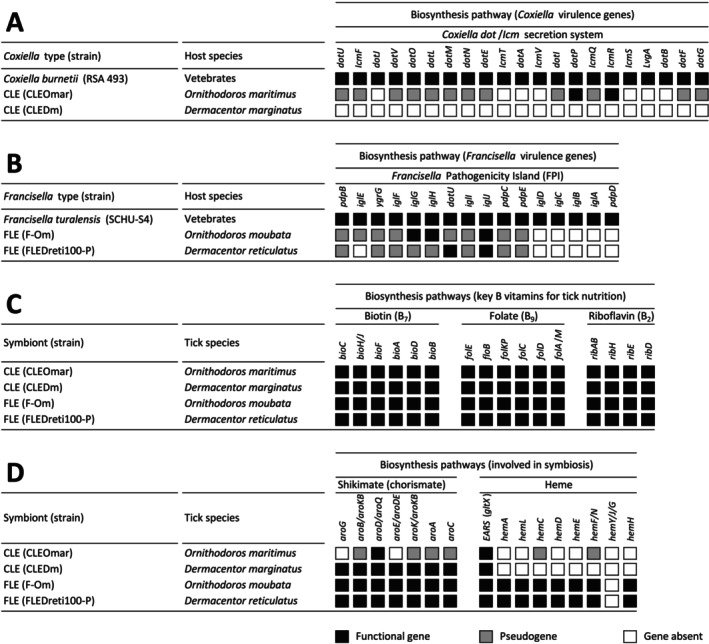
Conservation level of biosynthetic pathways in CLE and FLE genomes. (A) Pathogenic features of *Coxiella* genomes showing the conservation level of the *Dot*/*Icm* secretion system genes. (B) Pathogenic features of *Francisella* genomes showing the conservation level of the *Francisella* Pathogenicity Island (FPI) genes. (C) Biosynthetic pathways of CLE and FLE for the three key B vitamins involved in nutritional symbiosis with ticks. (D) Biosynthetic pathways potentially involved in symbiotic interactions with ticks. Black squares, functional genes; grey squares, pseudogenes; white squares, missing genes.

CLEOmar and CLEDm clustered within a well‐supported clade alongside other CLE genomes and *C. burnetii* in the Legionellales order (Figure S2), similar to the clade formed by F‐Om, FLEDreti100‐P, other FLE genomes, and *F. tularensis* in the Thiotrichales order (Figure S3). CLE and FLE exhibited characteristics of ongoing genome reduction, though at different stages (Table S4). CLEOmar was found to have a relatively larger genome (1.67 Mb) than CLEDm (0.9 Mb), although both fell within the typical size range observed in CLE (0.6–1.8 Mb) from other tick species (Santos‐Garcia et al. 2023; Guizzo et al. 2017; Gottlieb et al. 2015; Smith et al. 2015). In contrast, F‐Om (1.56 Mb) and FLEDreti100‐P (1.63 Mb) were more similar in size, comparable to other FLE genomes (~1.56 Mb) (Gerhart et al. 2018, 2016). Both CLE and FLE genomes displayed substantial reductions compared to their pathogenic relatives, *C. burnetii* (2 Mb) and *F. tularensis* (1.9 Mb), respectively. This reduction notably impacted virulence genes (Figure 4A,B). In the CLEOmar and CLEDm genomes, genes encoding the *Dot*/*Icm* secretion system in *C. burnetii* were either pseudogenized or missing. Similarly, in the F‐Om and FLEDreti100‐P genomes, most of the virulence genes of *F. tularensis* , including those within the *Francisella* Pathogenicity Island (FPI), were pseudogenized or absent. However, as some of these genomes are fragmented and disrupted across several contigs, any absent genes may also potentially be located within a genomic gap.

Despite evidence of genomic reduction, the CLE and FLE genomes still encoded most of the major pathways for B vitamins and cofactor biosynthesis (Figure 4C). Complete pathways for the biosynthesis of the three key B vitamins (biotin, riboflavin, and folate) were found to be conserved in CLEOmar, CLEDm, F‐Om, and FLEDreti100‐P. However, the pathways for other B vitamins had varying levels of completeness across genomes (Figure S4). For instance, for the biosynthesis pathway for nicotinate (B_3_), F‐Om and FLEDreti100‐P do not have any intact gene, whereas CLEOmar and CLEDm retained functional copies of most genes. Similarly, most genes involved in the biosynthetic pathway for pyridoxine (B_6_) were absent in F‐Om and FLEDreti100‐P, but all were present in CLEOmar and CLEDm.

Additionally, CLEDm, F‐Om, and FLEDreti100‐P consistently contained analogous complete shikimate pathways for chorismate biosynthesis (Figure 4D). These pathways, involved in the production of the tick tryptophan and serotonin, are complete in the CLEDm, F‐Om, and FLEDreti100‐P genomes and are similar to one previously identified in the CLE associated with the Asian longhorned tick *H. longicornis* (Zhong et al. 2021). However, the shikimate pathway in the CLEOmar genome was largely incomplete, with missing genes and pseudogenes. A key distinction between CLE and FLE genomes was their potential for heme production: F‐Om and FLEDreti100‐P were found to have most of the genes required for heme biosynthesis, while CLEOmar and CLEDm did not (Figure 4D). However, all four genomes, including CLEOmar and CLEDm, contained the *gltX* gene, which participates in heme biosynthesis by activating glutamate as a substrate. In F‐Om and FLEDreti100‐P, only one gene (*HemY*/*G*/*J*, encoding protoporphyrinogen oxidase) was missing (Figure 4D) though it is also absent in many bacteria with a similar heme biosynthesis pathway (Panek and O'Brian 2002). Its function could be compensated by another gene, particularly *HemN* (Panek and O'Brian 2002), indicating that F‐Om and FLEDreti100‐P might still be capable of producing heme without *HemY*/*G*/*J*.

## 4 Discussion

### 4.1 Analogous Symbiotic Interactions of CLE and FLE With Ticks

In this study, we demonstrate that two nutritional symbionts, CLE and FLE, have independently converged on analogous interactions with two tick genera, *Ornithodoros* and *Dermacentor*, belonging to two major tick families, Argasidae and Ixodidae. As observed in other tick genera (Gerhart et al. 2018, 2016; Santos‐Garcia et al. 2023; Duron et al. 2018; Guizzo et al. 2017; Gottlieb et al. 2015; Nardi et al. 2021; Smith et al. 2015), the CLE and FLE associated with *Ornithodoros* and *Dermacentor* species consistently possess complete biosynthesis pathways for the same core set of B vitamins (biotin, riboflavin, and folate) necessary for tick growth and survival (Duron and Gottlieb 2020). Here, our observations further reveal that the evolutionary convergence of CLE and FLE encompasses broader symbiotic traits: the two symbionts exhibit similar tissue distributions in their tick hosts, occupy similar intracellular (vacuolar) niches, and, although their genomes are largely pseudogenized, most of them have conserved intact analogous shikimate pathways for chorismate biosynthesis. However, interestingly, CLE and FLE differ in their capacity to biosynthesize other B vitamins, but also heme, an iron‐containing molecule that plays a crucial role in many metabolic processes of ticks, including oxygen transport and cellular respiration.

### 4.2 Similar Tissue Tropism of Nutritional Symbionts Across Tick Species

CLE and FLE have independently evolved similar mechanisms to stabilise their interactions with ticks through specific colonisation of ovaries and Malpighian tubules. Infection of the ovaries allows the maternal transmission of CLE and FLE into developing oocytes, ensuring their presence in nearly 100% of offspring, as previously observed in several tick species (Lalzar et al. 2012; Machado‐Ferreira et al. 2011; Duron, Noël, et al. 2015; Duron et al. 2018). Their high density in Malpighian tubules supports their nutritional role, as this organ is involved in excretion and osmoregulation and is located close to the gut (Duron and Gottlieb 2020). Like ticks, bed bugs, lice, and tsetse flies also host their B vitamin‐provisioning symbionts in organs that are part of, or connected to, the digestive tract, suggesting that nutritional symbionts commonly utilise metabolites excreted by their arthropod hosts to produce B vitamins (Duron and Gottlieb 2020). In most obligate blood feeders, B vitamin‐provisioning symbionts are housed in a specialised symbiotic organ, called a bacteriome, composed of clusters of specialised giant cells known as bacteriocytes (Duron and Gottlieb 2020). However, neither a bacteriome nor bacteriocytes are apparent in ticks. The observed tissue distribution of CLE and FLE suggests that Malpighian tubules may represent an equivalent of a bacteriome in the tick symbiotic systems. In this context, Malpighian tubules are integral components of the symbiotic relationship by providing a dedicated environment for CLE and FLE, potentially facilitating and regulating their functional interactions with ticks. The similarities in FLE and CLE tissue localisation may also arise from intrinsic tick host factors, particularly immune modulation and symbiont control. Shared ancestral mechanisms (potentially conserved from tick ancestors already engaged in nutritional symbiosis) may restrict symbionts to specific tissues, balancing immune tolerance with the nutritional benefits they provide. In some insects, bacteriocytes exhibit distinct immune profiles that enable symbiont maintenance while preventing colonisation of other tissues (Login et al. 2011; Anselme et al. 2008; Whittle et al. 2021). This compartmentalisation reflects a balance between immune tolerance and containment, potentially involving downregulation of antimicrobial responses and enrichment of key nutrients within bacteriocytes (Anselme et al. 2008).

### 4.3 Conserved Biosynthesis Pathways Supporting Nutritional Symbiosis

Genomic evidence suggests that nutritional symbionts may supplement ticks with analogous essential cofactors beyond B vitamins. For example, the chorismate biosynthesis pathways are present in most, although not all, CLE and FLE genomes. This conservation implies that most CLE and FLE may similarly provide tryptophan and increase serotonin production in their respective tick hosts, potentially stimulating feeding behaviour and regulating blood intake, as observed with the CLE of *H. longicornis* (Zhong et al. 2021). Therefore, both CLE and FLE could play a pivotal role as key partners for ticks, potentially influencing their feeding behaviour. However, FLE possesses the capability to biosynthesize heme, a metabolic function that is absent in CLE. Ticks are among the few eukaryotes that lack most of the genes necessary for heme synthesis and metabolism (Jia et al. 2020; Gulia‐Nuss et al. 2016). While vertebrate blood is typically considered the primary heme source for ticks (Jia et al. 2020; Gulia‐Nuss et al. 2016; Perner et al. 2016), the presence of heme synthesis pathways in FLE genomes suggests that these symbionts may play an additional role as a source of endogenous heme. Notably, ticks have evolved adaptations to survive prolonged periods (ranging from several months to years) without feeding (Jia et al. 2020; Gulia‐Nuss et al. 2016), and FLE‐produced heme may provide a crucial advantage during these fasting periods.

### 4.4 Intracellular Niches and Preadaptations to Nutritional Symbiosis

The intracellular niches occupied by CLE and FLE exhibit remarkable similarities. Both symbionts form dense clusters of bacteria within membrane‐bound vacuoles in the cytoplasm of tick cells, a phenotype previously observed in the CLE of *R. turanicus* (Lalzar et al. 2014). These bacterial aggregates can fill the entire cytoplasm, with vacuoles of varying sizes containing dividing bacteria, confirming their role as replication compartments. These structures closely mirror the membrane‐bound vacuoles formed by their pathogenic relatives, *C. burnetii* and *F. tularensis* , which manipulate vertebrate cell machinery to sustain their replication (van Schaik et al. 2013; Petit and Lebreton 2022; Degabriel et al. 2023). However, the pseudogenization and loss of virulence genes in CLE and FLE, which occurred similarly but independently, further support the hypothesis of their specialised adaptation from pathogenic ancestors to mutualistic symbionts (Santos‐Garcia et al. 2023; Duron et al. 2018). This transition could have been facilitated by preadaptations in their genomes since the biosynthetic pathways for essential nutrients found in CLE and FLE are ancestral traits shared with *C. burnetii* and *F. tularensis* (Santos‐Garcia et al. 2023; Duron et al. 2018), and some, such as the biotin biosynthesis pathway, are crucial for the replication of *C. burnetii* and *F. tularensis* within vertebrate macrophages (van Schaik et al. 2013; Petit and Lebreton 2022; Degabriel et al. 2023). These pathways may have thus originally evolved for bacterial survival and reproduction in vertebrates but were later exploited for nutritional purposes in ticks. In this context, these analogous ancestral traits shared by the *Coxiella* and *Francisella* genera likely contributed to the current CLE and FLE evolutionary convergence.

## 5 Conclusion

Beyond the observed convergence of bacterial symbionts, the similarities in FLE and CLE tissue localization may also be driven by intrinsic tick factors, with symbionts and hosts co‐evolving to optimise mutualistic interactions. These patterns could then reflect adaptive trade‐offs, where symbionts are confined to niches that maximise metabolic exchange while minimising the physiological costs to the host. Investigating the host‐symbiont dialogue, including immune signalling and molecular recognition, could help determine whether this localization is primarily dictated by host constraints or symbiont adaptations, providing deeper insights into the evolution of obligate mutualism. The analogous symbiotic traits that CLE and FLE have independently evolved further support the hypothesis that CLE and FLE could potentially be functionally interchangeable to some extent (Duron and Gottlieb 2020; Binetruy et al. 2020). Indeed, phylogenetic evidence consistently shows that CLE and FLE have exhibited competitive dynamics throughout the evolutionary history of ticks, with instances of symbiont extinction and replacement events in several tick lineages (Duron et al. 2017; Binetruy et al. 2020). FLEs have frequently replaced CLEs in ancestral tick lineages, suggesting that FLEs may offer additional benefits (Duron et al. 2017; Binetruy et al. 2020), which could include enhanced tick fitness under specific ecological conditions or more efficient transmission across tick generations. These findings emphasise the importance of understanding the ecological and evolutionary pressures that drive the persistence and replacement of symbionts in ticks. Future studies focusing on the mechanisms underlying competition between CLE and FLEs will be crucial to uncovering the full extent of their roles in tick biology and their interactions with tick hosts.

## Author Contributions

**Noor Fattar:** investigation, writing – original draft, visualization, formal analysis, data curation. **Meriem Louni:** investigation, writing – original draft, methodology, visualization, formal analysis, data curation. **Marie Buysse:** conceptualization, investigation, writing – review and editing, formal analysis, supervision. **Anna Maria Floriano:** investigation, writing – original draft, writing – review and editing, formal analysis, data curation. **Joanne Bertaux:** investigation, methodology, validation, visualization, writing – review and editing, formal analysis, data curation, supervision, resources. **Anne Cantereau:** resources, supervision, data curation, formal analysis, methodology, validation, visualization, writing – review and editing, investigation. **Ana Rivero:** writing – review and editing, methodology, validation, data curation, supervision. **Marjorie Bruley:** investigation, supervision, writing – review and editing. **Karen D. McCoy:** resources, writing – review and editing. **Vincent Delafont:** writing – review and editing, validation, data curation, supervision. **Nathalie Boulanger:** writing – review and editing, resources. **Fabrice Vavre:** supervision, writing – review and editing, data curation, funding acquisition, project administration. **Didier Bouchon:** data curation, supervision, writing – review and editing, funding acquisition, project administration. **Olivier Duron:** funding acquisition, writing – review and editing, writing – original draft, data curation, supervision, resources, project administration, validation, conceptualization.

## Ethics Statement

Tick manipulation was performed in a Biosafety Level 2 insectarium according to the regulations established by the Ethical and Animal Welfare Committee of the institution where the experiments were conducted, complying with the European legislation.

## Conflicts of Interest

The authors declare no conflicts of interest.

## Supporting information


**Figure S1.** (A, B) TEM micrographs of *O. moubata* ovaries (stage III oocyte) showing vacuoles with dividing FLE (pink arrows).
**Figure S2.** Whole genome phylogenetic relationship of CLE genomes examined in this study (in bold), and representative CLE and *C. burnetii* genomes, using maximum likelihood (ML) estimations (*Berkiella aquae*: GCF_001431295.2; CLEAA: CP007541.1; CLEDm: GCF_907164955.1; CLEOA: GCA_019425495.1; CLEOmar: GCF_907164965.1; CLERsF23UF6: GCA_018687865.1; CLE AaGA: CP021379; CLE Aa C904: GCA_000815025.1; CLE_Dsil_JLds763: doi.org/10.5061/dryad.t76hdr80p; CLE_Dsil_ShXds131: doi.org/10.5061/dryad.t76hdr80p; CLE_Dsil_Sxds774: doi.org/10.5061/dryad.t76hdr80p; CLE_Hlon_ZJHl565: doi.org/10.5061/dryad.t76hdr80p; CLE_Rmic_Gzbm458: doi.org/10.5061/dryad.t76hdr80p; CLE_Rmic_HbRm536: doi.org/10.5061/dryad.t76hdr80p; CLE_Rmic_Ynbm454: doi.org/10.5061/dryad.t76hdr80p; CLE_Rsan_GXRs837: doi.org/10.5061/dryad.t76hdr80p; CLE_Rsan_GXRs865: doi.org/10.5061/dryad.t76hdr80p; CLE_Rsan_Gxrs444: doi.org/10.5061/dryad.t76hdr80p; CRS_CAT: CP024961.1; *C. burnetii* _HasiXJHA496: doi.org/10.5061/dryad.t76hdr80p; *C. burnetii* RSA493: NC_002971.4; *C. burnetii* XJHA499: doi.org/10.5061/dryad.t76hdr80p; CeAS UFV: CP033868.1; *Coxiella* CLE.RmD: GCA_002930125.1; *Coxiella* CLERM: GCA_002871095.1; *Coxiella* CLE CRt: GCA_001077715.1; *Coxiella* CLE Craf2019: CP064834.1). Phylogenetic relationships were inferred from a concatenated alignment of 283 single‐copy orthologs (276,830 unambiguously aligned bp), using GTR + I + G4 as the best‐fit model of sequence evolution. The numbers on each node represent the bootstrap support percentage with 1000 replicates. The scale bar is in units of substitution/site. The pipeline for phylogenetic inference used for phylogenetic inferences was developed using custom scripts in Python 3, Bash, and R, and is available on GitHub (https://github.com/annamariafloriano/EvoNutrSymb_Fattar_Louni). Briefly, the CLE dataset was filtered to exclude pseudogene sequences predicted by PseudoFinder before being used for phylogenetic analyses. The intact genes were translated into proteins and single‐copy orthologs (SCOs) were identified using OrthoFinder (v2.3.12) (Duron and Gottlieb 2020). The nucleotide sequences of the SCOs were recovered and aligned by codons with SeaView (v5.0.5) (Manzano‐Marín et al. 2015), and consequently concatenated. Finally, the most suitable evolutionary model was determined using ModelTest‐NG (v0.1.5) (Nikoh et al. 2014) based on the Akaike Information Criterion (AIC). Maximum likelihood (ML) trees were then inferred using RAxML (v8.2.12) (Sterkel et al. 2017) with 1000 bootstrap replicates.
**Figure S3.** Whole genome phylogenetic relationship of FLE genomes examined in this study (in bold), and representative FLE and *F. tularensis* genomes, using maximum likelihood (ML) estimations (*F. adeliensis* strain FDC440: GCF_003290445.1; *F. frigiditurris* strain CA97 1460: NZ_CP009654.1; *F. halioticida* strain DSM23729: GCF_002211785.1; *F. hispaniensis* strain 3523: GCF_000195555.1; *Francisella* FLEAm: LNCT01.1; FLE Dreti100P: pending; FLE Hasi NMGha312: doi.org/10.5061/dryad.t76hdr80p; FLE Hasi NMGha432: doi.org/10.5061/dryad.t76hdr80p; FLE Hasi XJHA498: doi.org/10.5061/dryad.t76hdr80p; FLE HmarESP: GCA_910592745.1; FLE HmarIL: GCA_910592815.1; FLE HmarIT: GCA_910592705.1; *Francisella* FLEOm: LVCE01.1; *F. marina* E95 16: GCF_008369785.1; *F. noatunensis* strain FSC1145: GCF_016605205.1; *F. opportunistica* isolate 142,155: GCF_003347095.1; *F. orientalis* LADL 07285A: GCF_000505725.1; *F. persica* ATCC VR 331: CP013022.1; *F. philomiragia* isolate 18,844: NZ_CP063138.1; *F. salimarina* strain CHUGAF75: GCF_018972105.1; *F. salina* strain TX07 7308: GCF_000219045.1; *F. tularensis* D9876: GCF_000833355.1; *F. tularensis* NIHB38: GCF_000833475.1; *F. uliginis* TX07 7310: GCF_001895265.1; FLE Fom: QAPC01.1). Phylogenetic relationships were inferred from a concatenated alignment of 438 single‐copy orthologs (414,485 unambiguously aligned bp), using GTR + I + G4 as the best‐fit model of sequence evolution. The numbers on each node represent the bootstrap support percentage with 1000 replicates. The scale bar is in units of substitution/site. We applied the same pipeline as CLEs for phylogenetic inference (see the description of Figure S2 for details).
**Figure S4.** Conservation level of biosynthetic pathways for other B vitamins in CLE and FLE genomes. Black squares, functional genes; grey squares, pseudogenes; white squares, missing genes.
**Table S1.** List and origin of tick specimens examined in this study.
**Table S2.** List of genes and primers used for real‐time quantitative polymerase chain reaction (qPCR) assays.
**Table S3.** List of 16S rDNA probes used for fluorescence in situ hybridization (FISH).
**Table S4.** Genomic features of CLEOmar, CLEDm, F‐Om, and FLEDreti100‐P.
**Table S5.** Statistical models used for symbiont densities in tick organs.

**Video S1.** A three‐dimensional FISH reconstruction showing the cytoplasmic distribution of CLE (green) within the ovarian cells of the *O. maritimus* tick. The CLE symbionts are often enclosed in small vacuoles, giving the cytoplasmic content a cottony appearance. These vacuoles are dispersed throughout the depth of cells.

## Data Availability

The qPCR dataset for symbiont quantification in ticks is available on GitHub (https://github.com/annamariafloriano/EvoNutrSymb_Fattar_Louni).
