# Supplementary material for: Evolutionary Convergence of Nutritional Symbionts in Ticks
Source: Environ Microbiol Rep. 2025 Jun 10;17(3):e70120. doi: 10.1111/1758-2229.70120 (PMC12152204; doi:10.1111/1758-2229.70120)
Supplement: Supplementary file 1 — Figure S1. (A, B) TEM micrographs of O. moubata ovaries (stage III oocyte) showing vacuoles with dividing FLE (pink arrows). Figure S2. Whole genome phylogenetic relationship of CLE genomes examined in this study (in bold), and representative CLE and C. burnetii genomes, using maximum likelihood (ML) estimations (Berkiella aquae: GCF_001431295.2; CLEAA: CP007541.1; CLEDm: GCF_907164955.1; CLEOA: GCA_019425495.1; CLEOmar: GCF_907164965.1; CLERsF23UF6: GCA_018687865.1; CLE AaGA: CP021379; CLE Aa C904: GCA_000815025.1; CLE_Dsil_JLds763: doi.org/10.5061/dryad.t76hdr80p; CLE_Dsil_ShXds131: doi.org/10.5061/dryad.t76hdr80p; CLE_Dsil_Sxds774: doi.org/10.5061/dryad.t76hdr80p; CLE_Hlon_ZJHl565: doi.org/10.5061/dryad.t76hdr80p; CLE_Rmic_Gzbm458: doi.org/10.5061/dryad.t76hdr80p; CLE_Rmic_HbRm536: doi.org/10.5061/dryad.t76hdr80p; CLE_Rmic_Ynbm454: doi.org/10.5061/dryad.t76hdr80p; CLE_Rsan_GXRs837: doi.org/10.5061/dryad.t76hdr80p; CLE_Rsan_GXRs865: doi.org/10.5061/dryad.t76hdr80p; CLE_Rsan_Gxrs444: doi.org/10.5061/dryad.t76hdr80p; CRS_CAT: CP024961.1; C. burnetii _HasiXJHA496: doi.org/10.5061/dryad.t76hdr80p; C. burnetii RSA493: NC_002971.4; C. burnetii XJHA499: doi.org/10.5061/dryad.t76hdr80p; CeAS UFV: CP033868.1; Coxiella CLE.RmD: GCA_002930125.1; Coxiella CLERM: GCA_002871095.1; Coxiella CLE CRt: GCA_001077715.1; Coxiella CLE Craf2019: CP064834.1). Phylogenetic relationships were inferred from a concatenated alignment of 283 single‐copy orthologs (276,830 unambiguously aligned bp), using GTR + I + G4 as the best‐fit model of sequence evolution. The numbers on each node represent the bootstrap support percentage with 1000 replicates. The scale bar is in units of substitution/site. The pipeline for phylogenetic inference used for phylogenetic inferences was developed using custom scripts in Python 3, Bash, and R, and is available on GitHub (https://github.com/annamariafloriano/EvoNutrSymb_Fattar_Louni). Briefly, the CLE dataset was filtered to exclude pseudogene sequences predi [file EMI4-17-e70120-s002.docx]

**Supplementary materials**

**Evolutionary convergence of nutritional symbionts in ticks**

## **Table S1.** List and origin of tick specimens examined in this study.

| **Tick species** | **Nutritional symbiont** | **Sampling site** | **Environment** |
| --- | --- | --- | --- |
| *Ornithodoros moubata* | FLE | Lab colony (Neuchâtel strain), France (2024) | Lab colonny |
| *Ornithodoros maritimus* | CLE | Carteau Island, France (2024) | Sea bird nest |
| *Dermacentor marginatus* | CLE | Saint Gely du Fesc, France (2024) | Vegetation |
| *Dermacentor reticulatus* | FLE | Strasbourg, France (2024) | Vegetation |

**Table S2.** List of genes and primers used for real-time quantitative polymerase chain reaction (qPCR) assays.

| Target | Gene | Name | Sequence (5’-3’) | Reference |
| --- | --- | --- | --- | --- |
| Ticks | *actin* | Omou_actqF2 | CGGTATTGCCGACCGTATGC | [1] |
|  |  | Omou_actqR1 | CTCCCTGTCCACCTTCCAGC | [1] |
| FLE (*O. moubata*, *D. reticulatus*) | *rpoB* | Fra_rpoBqF | GTGGTGTACCTATTGCTACG | [1] |
|  |  | Fra_rpoBqR | GCTTACCAGTACGACCATCA | [1] |
| CLE (*O. maritimus*) | *rpoB* | CLE_rpoBqF | ATTGTACGATGGTCGAACC | This study |
|  |  | CLE_rpoBqR | CACGCGCGCTCCACAGGATC | This study |
| CLE (*D. marginatus*) | *rpoB* | CLEDmar-rpoBqF | TCTATACGATGGGCGAACC | This study |
|  |  | CLEDmar-rpoBqR | GAACCCGTAGAACGAGCGTG | This study |

**References**

[1] Duron O, Morel O, Noël V, Buysse M, Binetruy F, Lancelot R, et al. 2018 Tick-bacteria mutualism depends on B vitamin synthesis pathways. *Curr. Biol.* *28*, 1896-1902

## **Table S3.** List of 16S rDNA probes used for fluorescence in situ hybridization (FISH).

| Target | Name | Sequence (5’-3’) | Reference |
| --- | --- | --- | --- |
| CLE (*O. maritimus*) | Cox_C2 (probe) | CTTCTATGGGTAACGTCAGCGCC | This study |
| CLE (*D. marginatus*) | Cox_C2_Dmar (probe) | CTTCTGTAGGTAACGTCAACGCT | This study |
| FLE (*O. moubata*, *D. reticulatus*) | Frla_F2 (probe) | ATTCTTTGGGTAACGTCCCTC | This study |
|  | Frla_F2H1 (helper for the Frla_F2 probe) | CTCAAGGCTATTAACCTCA | This study |
|  | Frla_F2H2 (helper for the Frla_F2 probe) | CACGGAGTTAGCCGGTGCT | This study |

**Table S4.** Genomic features of CLEOmar, CLEDm, F-Om, and FLEDreti100-P.

| Bacteria | GenBank | Genome size (bp) | GC content | Completeness | Redundancy | Weighted completeness | Weighted redundancy | Contigs number | N50 (bp) |
| --- | --- | --- | --- | --- | --- | --- | --- | --- | --- |
| *Coxiella* |  |  |  |  |  |  |  |  |  |
| CLEDm | GCF_907164955.1 | 901,206 | 34.98 | 0.9238 | 1.0000 | 0.8838 | 1.0 | 1 | 901,206 |
| CLEOmar | GCA_907164965.1 | 1,671,336 | 41.65 | 0.9905 | 1.0000 | 0.9814 | 1.0 | 112 | 24,462 |
| *Coxiella burnetii* (RSA 493 strain) | NC_002971.4 | 1,995,488 | 42.66 | 1.0000 | 1.0000 | 1.0 | 1.0 | 1 | 1,995,488 |
| *Francisella* |  |  |  |  |  |  |  |  |  |
| FLEOm | QAPC00000000 | 1,564,190 | 31.7 | 0.9905 | 1.0096 | 0.9918 | 1.0 | 8 | 395,819 |
| FLEDreti100-P | JBMWAK000000000 | 1,627,125 | 33.58 | 0.9714 | 1.2059 | 0.9357 | 1.1581 | 36 | 266,549 |
| *Francisella tularensis* (D9876 strain) | GCF000833355.1 | 1,870,206 | 32.48 | 0.9905 | 1.0192 | 1.0 | 1.0152 | 1 | 1,870,206 |

**Table S5.** Statistical models used for symbiont densities in tick organs.

| Tick species | Variable of interest | Response variable | Model # | Maximal model | Minimal model | R subrout |
| --- | --- | --- | --- | --- | --- | --- |
|  |  |  |  |  |  |  |
| All species | Symbiont density in ovaries vs in testes | logSymb | 1 | Species*Sex + (1 \| Indiv) | Species*Sex + (1 \| Indiv) | lm |
|  | Symbiont density in ovaries vs in other organs | logSymb | 2 | SexOrgan + (1 \| Indiv) | SexOrgan + (1 \| Indiv) | glmer |
|  | Symbiont density (CLE vs FLE) in all organs | logSymb | 3 | Symbiote*SexOrgan + (1\| Indiv) | Symbiote*SexOrgan + (1\| Indiv) | glmer |

**Legend of supplementary video and figures**

**Video S1.** A three-dimensional FISH reconstruction showing the cytoplasmic distribution of CLE (green) within the ovarian cells of the O. maritimus tick. The CLE symbionts are often enclosed in small vacuoles, giving the cytoplasmic content a cottony appearance. These vacuoles are dispersed throughout the depth of cells.

**Figure S1.** (A, B) TEM micrographs of *O. moubata* ovaries (stage III oocyte) showing vacuoles with dividing FLE (pink arrows).

**Figure S2.** Whole genome phylogenetic relationship of CLE genomes examined in this study (in bold), and representative CLE and *C. burnetii* genomes, using maximum likelihood (ML) estimations (*Berkiella aquae*: GCF_001431295.2; CLEAA: CP007541.1; CLEDm: GCF_907164955.1; CLEOA: GCA_019425495.1; CLEOmar: GCF_907164965.1; CLERsF23UF6: GCA_018687865.1; CLE AaGA: CP021379; CLE Aa C904: GCA_000815025.1; CLE_Dsil_JLds763: doi.org/10.5061/dryad.t76hdr80p; CLE_Dsil_ShXds131: doi.org/10.5061/dryad.t76hdr80p; CLE_Dsil_Sxds774: doi.org/10.5061/dryad.t76hdr80p; CLE_Hlon_ZJHl565: doi.org/10.5061/dryad.t76hdr80p; CLE_Rmic_Gzbm458: doi.org/10.5061/dryad.t76hdr80p; CLE_Rmic_HbRm536: doi.org/10.5061/dryad.t76hdr80p; CLE_Rmic_Ynbm454: doi.org/10.5061/dryad.t76hdr80p; CLE_Rsan_GXRs837: doi.org/10.5061/dryad.t76hdr80p; CLE_Rsan_GXRs865: doi.org/10.5061/dryad.t76hdr80p; CLE_Rsan_Gxrs444: doi.org/10.5061/dryad.t76hdr80p; CRS_CAT: CP024961.1; *C. burnetii*_HasiXJHA496: doi.org/10.5061/dryad.t76hdr80p; *C. burnetii* RSA493: NC_002971.4; *C. burnetii* XJHA499: doi.org/10.5061/dryad.t76hdr80p; CeAS UFV: CP033868.1; *Coxiella* CLE.RmD: GCA_002930125.1; *Coxiella* CLERM: GCA_002871095.1 ; *Coxiella* CLE CRt: GCA_001077715.1 ; *Coxiella* CLE Craf2019: CP064834.1). Phylogenetic relationships were inferred from a concatenated alignment of 283 single-copy orthologs (276,830 unambiguously aligned bp), using GTR+I+G4 as the best-fit model of sequence evolution. The numbers on each node represent the bootstrap support percentage with 1,000 replicates. The scale bar is in units of substitution/site. The pipeline for phylogenetic inference used for phylogenetic inferences was developed using custom scripts in Python 3, Bash, and R, and is available on GitHub (<https://github.com/annamariafloriano/EvoNutrSymb_Fattar_Louni>). Briefly, the CLE dataset was filtered to exclude pseudogene sequences predicted by PseudoFinder before being used for phylogenetic analyses. The intact genes were translated into proteins and single-copy orthologs (SCOs) were identified using OrthoFinder (v2.3.12) [1]. The nucleotide sequences of the SCOs were recovered and aligned by codons with SeaView (v5.0.5) [2], and consequently concatenated. Finally, the most suitable evolutionary model was determined using ModelTest-NG (v0.1.5) [3] based on the Akaike Information Criterion (AIC). Maximum likelihood (ML) trees were then inferred using RAxML (v8.2.12) [4] with 1,000 bootstrap replicates.

**References**

[1] Emms DM, Kelly S. 2019 OrthoFinder: phylogenetic orthology inference for comparative genomics. *Genome Biol.* **20**, 238 (doi: 10.1186/s13059-019-1832-y)

[2] Gouy M, Tannier E, Comte N, Parsons DP. 2021 Seaview version 5: A multiplatform software for multiple sequence alignment, molecular phylogenetic analyses, and tree reconciliation. In *Multiple Sequence Alignment: Methods and Protocols* (ed K Katoh), pp 240-260. New York, NY: Springer (doi: 10.1007/978-1-0716-1036-7_15)

[3] Darriba D, Posada D, Kozlov AM, Stamatakis A, Morel B, Flouri T. 2020 ModelTest-NG: A new and scalable tool for the selection of DNA and protein evolutionary models. *Mol. Biol. Evol.* **37**, 291–294

[4] Stamatakis A. 2014 RAxML version 8: a tool for phylogenetic analysis and post-analysis of large phylogenies. *Bioinformatics* **30**, 1312–1313

**Figure S3.** Whole genome phylogenetic relationship of FLE genomes examined in this study (in bold), and representative FLE and *F. tularensis* genomes, using maximum likelihood (ML) estimations (*F. adeliensis* strain FDC440: GCF_003290445.1; *F. frigiditurris* strain CA97 1460: NZ_CP009654.1; *F. halioticida* strain DSM23729: GCF_002211785.1; *F. hispaniensis* strain 3523: GCF_000195555.1; *Francisella* FLEAm: LNCT01.1; FLE Dreti100P: pending; FLE Hasi NMGha312: doi.org/10.5061/dryad.t76hdr80p; FLE Hasi NMGha432: doi.org/10.5061/dryad.t76hdr80p; FLE Hasi XJHA498: doi.org/10.5061/dryad.t76hdr80p; FLE HmarESP: GCA_910592745.1; FLE HmarIL: GCA_910592815.1; FLE HmarIT: GCA_910592705.1; *Francisella* FLEOm: LVCE01.1; *F. marina* E95 16: GCF_008369785.1; *F. noatunensis* strain FSC1145: GCF_016605205.1; *F. opportunistica* isolate 14 2155: GCF_003347095.1; *F. orientalis* LADL 07 285A: GCF_000505725.1; *F. persica* ATCC VR 331: CP013022.1; *F. philomiragia* isolate 18844: NZ_CP063138.1; *F. salimarina* strain CHUGAF75: GCF_018972105.1; *F. salina* strain TX07 7308: GCF_000219045.1; *F. tularensis* D9876: GCF_000833355.1; *F. tularensis* NIHB38: GCF_000833475.1; *F. uliginis* TX07 7310: GCF_001895265.1; FLE Fom: QAPC01.1). Phylogenetic relationships were inferred from a concatenated alignment of 438 single-copy orthologs (414,485 unambiguously aligned bp), using GTR+I+G4 as the best-fit model of sequence evolution. The numbers on each node represent the bootstrap support percentage with 1,000 replicates. The scale bar is in units of substitution/site. We applied the same pipeline as CLEs for phylogenetic inference (see the description of Figure S2 for details).

**Figure S4.** Conservation level of biosynthetic pathways for other B vitamins in CLE and FLE genomes. Black squares, functional genes; gray squares, pseudogenes; white squares, missing genes.


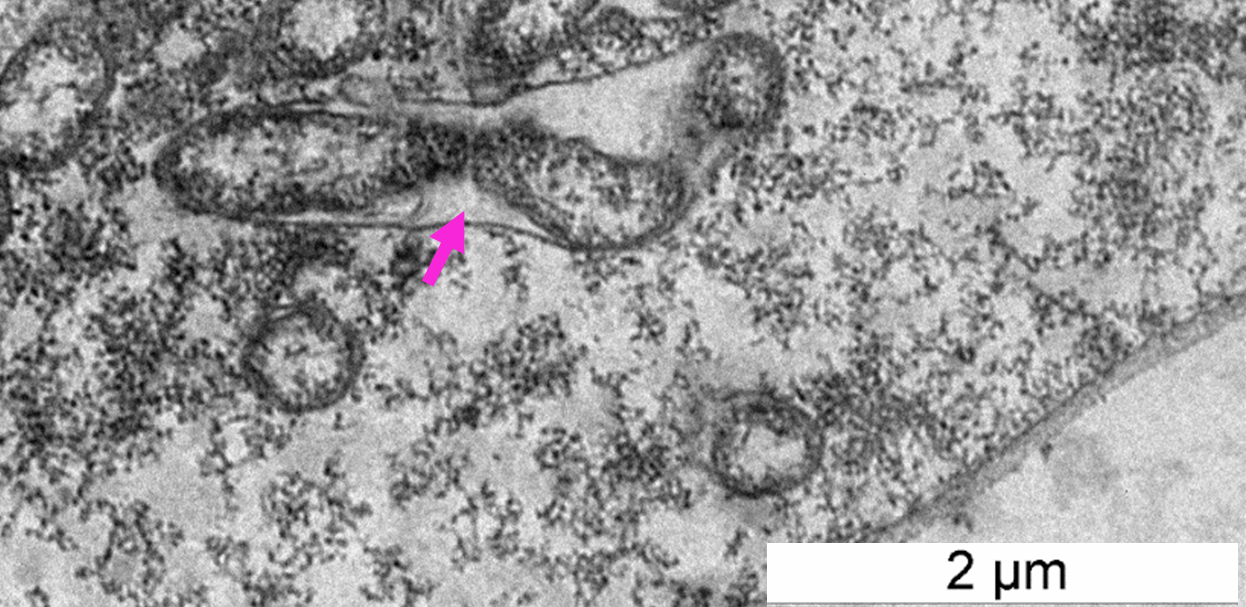


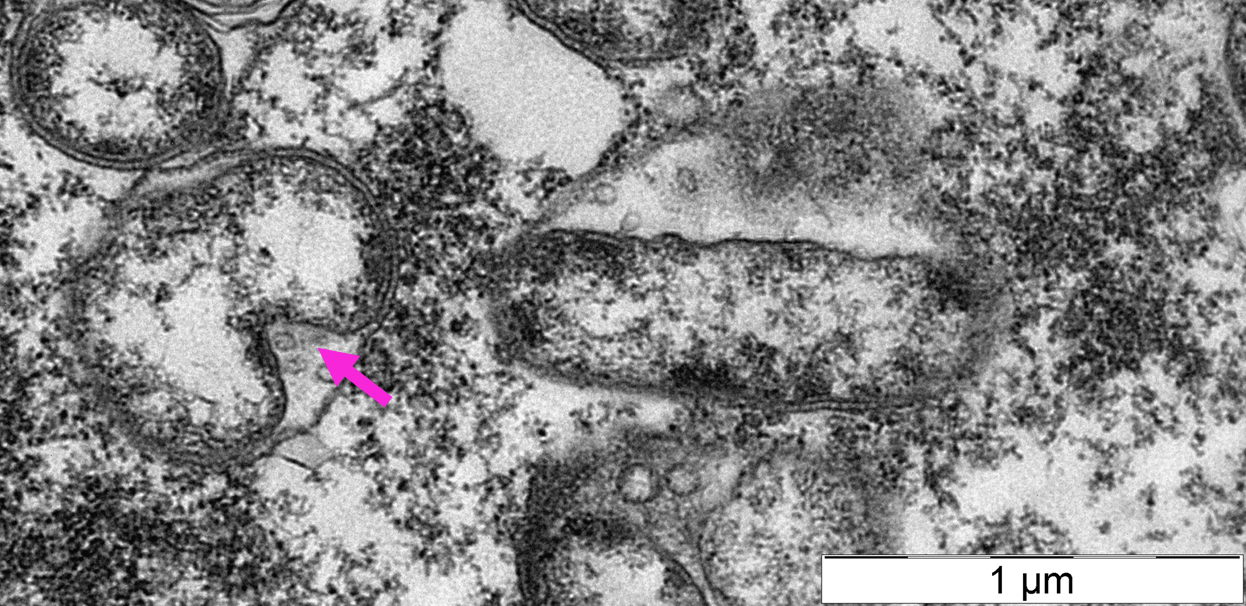


**Figure S1**

**Figure S2**

**Figure S3**

**Figure S4**
